# Hemichorea as the First and Sole Manifestation in Lupus: Case-Based Review

**DOI:** 10.31138/mjr.180323.haf

**Published:** 2023-09-15

**Authors:** Harikrishnan Gangadharan, Rahul Peter, Vineetha VS, Varghese Punnoose, Josemon George

**Affiliations:** 1 Unit of Rheumatology, Department of Internal Medicine, Government Medical College Kottayam, Kerala, India,; 2 Department of Internal Medicine, Government Medical College Kottayam, Kerala, India,; 3 Department of Neurology, Government Medical College Thiruvananthapuram, Kerala, India,; 4 Department of Psychiatry, Government Medical College Kottayam, Kerala, India

**Keywords:** SLE, chorea, neuropsychiatric lupus, movement disorder

## INTRODUCTION

Neuropsychiatric manifestations can be seen in 12–95% of SLE patients and can affect both the central as well as peripheral nervous system.^1^ Movement disorder is a well-defined clinical syndrome of NPSLE, and it is unusual to find movement disorders as the first and only manifestation of SLE. There is a paucity of literature on chorea as the first and sole manifestation of lupus. We describe a young girl who presented with hemichorea as the first and sole manifestation of SLE. She also tested positive for lupus anticoagulant. We have also done a brief literature review on chorea as the presenting feature of SLE. Our case study adds to the limited available literature on this rare presentation of SLE and highlights the importance of suspecting lupus in any patient with chorea.

## CASE HISTORY

A 17- year-old girl from South India presented with abnormal involuntary movements of the left hand and foot of 1-month duration. The movements were brief-lasting, irregular, purposeless, and used to disappear during sleep. There was no involvement of the proximal part of limbs, face, tongue, or altered consciousness during the abnormal movement. There was no prior history suggestive of acute rheumatic fever, thyroid disease, diabetes, jaundice, or intake of any drugs. There was no history of oral ulcers, photosensitivity, joint pain, frothing of urine, or decreased urine output. There was no family history of involuntary movements. Upon examination, she had normal sensorium and vital signs. There were irregular involuntary non-rhythmic brief lasting jerky movements confined to the left hand and foot suggestive of left hemichorea (**Supplementary Video 1** https://youtube.com/shorts/w1CyXMeGSzE). The rest of the examination of the neurological and other systems was normal. Routine blood tests were normal except for raised erythrocyte sedimentation rate (ESR)- 50 mm/hour (0–15mm/hour), and prolonged activated partial thromboplastin time (APTT)- 48 seconds (0–30 seconds). Considering her age, raised ESR and prolonged APTT, SLE with anti-phospholipid antibody syndrome was strongly suspected and further evaluation revealed positive anti-nuclear antibody (ANA) by indirect immunofluorescence (IF)- 3+ nuclear homogenous pattern in 1:100 dilution, raised anti-double stranded DNA (Anti dsDNA)- 125 IU/ml (0–25 IU/ml), low C3- 47mg/dl (90-180mg/dl), low C4- 3.07mg/dl (10–40mg/dl) and positive lupus anticoagulant 1.94 (0–1.3). Magnetic resonance imaging (MRI) Brain was normal and Digital subtraction angiography (DSA) did not show any evidence of CNS vasculitis. Thus, a final diagnosis of SLE with neuropsychiatric lupus in the form of left hemichorea and secondary antiphospholipid antibody syndrome was made. She was treated with low- dose oral prednisolone (7.5 mg daily), hydroxychloroquine, and tetrabenazine (dopamine antagonist). The patient showed a good response and on follow-up at 1 month, the hemichorea had resolved (**Supplementary Video 2** https://youtube.com/shorts/nfCVq_z2A3k). After 3 months, her lupus anticoagulant was checked again, and it remained positive. The patient was off dopamine antagonist and on 2.5 mg oral prednisolone and hydroxychloroquine (300mg per day) and continued to be in clinical remission. However, she discontinued her medicines and was lost to follow-up for 9 months. After 9 months she returned to us with inflammatory polyarthritis of bilateral knee, elbow, and ankle, and a vasculitic rash of bilateral palms and dorsum of the foot. She was managed with 0.5mg/kg oral prednisolone and hydroxychloroquine and the symptoms resolved. She is currently on a tapering dose of oral corticosteroids.

## DISCUSSION

SLE is the prototypical multi-system autoimmune disease with neurological involvement ranging from non-specific headache to life-threatening demyelination syndromes. The American College of Rheumatology (ACR) nomenclature for neuropsychiatry syndromes in SLE includes 12 central nervous system syndromes and 7 peripheral nervous system syndromes.^2^ The classification of pathogenic mechanisms responsible for various neuropsychiatry syndromes into ischemic and neuroinflammatory may be an oversimplification of this clinically challenging entity. Due to the heterogeneity in the inclusion criteria and methodology, there is a wide variation in the frequency of NPSLE in various studies. Mild cognitive dysfunction and mood disorders are frequently seen as manifestations of NPSLE (6–80%) whereas movement disorders including chorea are extremely rare (< 1% of SLE patients).^3^

Chorea as the initial and only manifestation of SLE is extremely uncommon. We followed the search strategy suggested by Gasparyan et al.^4^ for writing a case-based biomedical review with a systematic approach. As a part of comprehensive literature review, we searched PubMed (MedLine), Scopus and Directory of Open Access Journals (DOAJ) databases from their inception to 3^rd^ June 2023. A PubMed search using the keywords “CHOREA” OR “HEMICHOREA” AND “LUPUS ERYTHEMATOSUS, SYSTEMIC” yielded 244 results. A Scopus search using the keywords “CHOREA” OR “HEMICHOREA” AND “SYSTEMIC LUPUS ERYTHEMATOSUS” yielded 445 results and a search in Directory of Open Access Journals (DOAJ) using the keywords CHOREA AND SYSTEMIC LUPUS ERYTHEMATOSUS yielded 19 results. After excluding duplicate articles, irrelevant articles, review articles, articles which did not have an English text, and articles which did not have a full text available, we were able to identify 24 articles comprising of case reports, case series and cohort studies describing a total of 64 patients where chorea was the first manifestation of SLE (**Table 1**).

**Table 1.** Summary of patients with chorea as the initial manifestation of SLE.

**Serial No/Reference No**	**Author**	**Type of study**	**Number of cases**	**Age of patient**	**Age at onset of chorea**	**Gender**	**Other features of SLE**	**Interval from onset of chorea to other symptoms of SLE**	**Presence of antiphospholipid antibody**	**Treatment**	**Outcome**
**1/5**	Arisaka et al.	Case report	1	10	10 years	F	Thrombocytopenia, malar rash, low C3, low C4, ANA +, anti dsDNA+	3 months	NA	Prednisolone, haloperidol	Resolution of chorea
**2/6**	Bruyn et al.	Critical review	11	-	18 years (mean age)	8 female, 3 male	Not mentioned	32 months	NA	Prednisolone, haloperidol	Resolution of chorea
**3/7**	Poil et al.	Case report	1	27	27	F	ANA+, anti-ds DNA +, anti Sm+, low C3	0	present	Aspirin, hydroxychloroquine	Resolution of chorea
**4/8**	Abdalla et al.	Case report	1	14	14	F	Thrombocytopenia, ANA +, Coombs test +, anti ds DNA +, haematuria	0	Present	Iv methylprednisolone, enoxaparin, hydroxychloroquine, mycophenolic acid	Resolution of chorea
**5/9**	Ariizumi et al.	Case report	1	68	68	F	Leukopenia, ANA+, anti ds DNA +, anti SSA+	0	Present	Haloperidol, aspirin	Resolution of chorea
**6/10**	Albishri et al.	Case report	1	15	15	F	ANA+, anti-ds DNA+, class 4 lupus nephritis	2 weeks	Absent	Haloperidol, Mycophenolate mofetil, prednisolone	Mild persistence of chorea
**7/11**	Kakehasi et al.	Case report	1	36	36	F	Oral ulcer, arthritis, anaemia, thrombocytopenia, ANA+, anti SM+, anti ds DNA +	1 week	Absent	Prednisolone, phenobarbital, phenytoin, clonazepam	Resolution of chorea
**8/12**	Kukla et al.	Case report	1	11	11	M	Fever, leukopenia, thrombocytopenia, ANA+, Anti ds DNA +, Coombs +, low C3, C4, cutaneous vasculitis, nephritis	1 week	NA	Haloperidol, prednisolone	Resolution of chorea
**9/13**	Torregiani et al.	Case report	1	12	12	M	ANA+, anti-ds DNA+, Coombs +, arthritis, class 3 lupus nephritis, deep vein thrombosis	0	present	Intravenous methylprednisolone pulse followed by oral prednisolone	Resolution of chorea
**12/15**	Reiner et al.	Retrospective cohort	28	20.6 (mean age)	Not mentioned	25 F 3 M	ANA+, thrombocytopenia, haemolytic anaemia, lymphopenia arthritis, headache, stroke	0	24/26 patients (92%)	Corticosteroid, aspirin, benzodiazepine, IVIG, anti- convulsant, plasma exchange.	Resolution in majority of patients. 8 patients had relapse of chorea.
**10/16**	Carvallo et al.	Case series	3	27.3 (mean age)	27.3 (mean age)	F	ANA+, Anti dsDNA+, Lymphopenia, alopecia, nephritis, psychosis, seizures	2–4 months	Positive IgM Anti-cardiolipin antibody	Haloperidol, corticosteroid, valproic acid, ziprasidone, cyclophosphamide	Resolution
**11/18**	Medeiros et al.	Case report	1	20	20	F	ANA+, Anti dsDNA+, low C3, C4, leukocytoclastic vasculitis, class 4 lupus nephritis, malar rash, arthritis	0	negative	Intravenous methylprednisolone pulse dose followed by oral prednisolone, mycophenolate mofetil, haloperidol	Resolution
**13/19**	Ostovan et al.	Case report	1	27	27	M	Central retinal vein and artery occlusion, thrombocytopenia, ANA+, Anti ds DNA+, nephrotic range proteinuria	0	positive	Anticoagulation, immunosuppression	resolution
**14/20**	Fermaglich et al.	Case report	1	18	11	F	ANA+, anti-ds DNA +, thrombocytopenia, nephritis	7 years	present	Haloperidol	resolution
**15/21**	Thomas et al.	Case report	1	22	22	F	Rash on extremities, psychosis, thrombocytopenia, ANA+, anti dsDNA+	0	NA	thiopropazate	resolution
**16/22**	Heilman et al.	Case report	1	21	15	F	Seizure, polyarthritis, nephritis	3 years	NA	Haloperidol, azathioprine	resolution
**17/23**	Khamashta et al.	Case report	1	22	17	F	Polyarthralgia, malar rash, fever, leukopenia, thrombocytopenia, ANA+, Anti dsDNA+. Anti Sm+	0	present	Methylprednisolone, cyclophosphamide	resolution
**18/24**	Groothuis et al.	Case report	1	7	7	M	Rash, fever, thrombocytopenia, ANA+, Anti dsDNA+, low C3, nephritis	9 days	NA	prednisolone	resolution
**19/25**	Loh et al.	Retrospective cohort	1	11	Not mentioned	F	Nephrotic syndrome	Not mentioned	NA	Haloperidol, prednisolone, cyclophosphamide	resolution
**20/26**	Sonu et al.	Case report	1	67	67	F	Anaemia, leukopenia, ANA+ anti ds DNA +. Low C3, low C4	0	absent	Tetrabenazine, olanzapine, hydroxychloroquine, methylprednisolone, cyclophosphamide	resolution
**21/27**	Allan et al.	Case report	1	14	14	M	ANA+, low C3, C4, arthritis, thrombocytopenia, rash, nephritis	16 months	present	Prednisolone, warfarin, hydroxychloroquine	resolution
**22/28**	Lusins et al.	Case series	2	41.5 (mean age)	41.5 (mean age)	F	ANA+	2 months	NA	Prednisolone, haloperidol	Persistence of chorea in 1 case, resolution of chorea in 2^nd^ case.
**23/29**	Olsen et al.	Case report	1	28	16	F	LE cell phenomenon positive, false positive VDRL, nephritis, peripheral neuropathy	12 years	NA	aspirin	resolution
**24/30**	Demir et al.	Case report	1	13	13	F	Anaemia, leukopenia, lymphopenia, thrombocytopenia, class 2 lupus nephritis, ANA+, Anti ds DNA+, low C3, C4	0	Absent	IVIG, pulse methyl prednisolone, cyclophosphamide, hydroxychloroquine	resolution
**25**	Present case	Case report	1	17	17	F	ANA+, Anti ds DNA+, low C3, C4, polyarthritis, vasculitic rash	0	Lupus anticoagulant positive	Oral prednisolone(7.5mg), hydroxychloroquine, tetrabenazine (dopamine antagonist)	resolution

F: female; M: male; ANA: Anti-nuclear antibody; Anti-ds DNA: Anti-double-stranded DNA; NA: not available.

Arisaka et al.^5^ described a 10- year- old girl who presented with chorea and 10 months later developed a clinical picture suggestive of SLE. The patient was initially thought to be having Sydenham’s chorea and was treated with penicillin and oral prednisolone. A mild reduction in the complement level was the only early clinical clue to the diagnosis of SLE in this patient. The authors have not mentioned the presence of antiphospholipid antibodies in this patient. In a critical analysis of 51 cases of chorea in SLE, Bruyn et al.^6^ found that chorea occurs early in the disease course of SLE and in 11 cases chorea was the initial manifestation. The authors have not described the association of chorea with the presence of antiphospholipid antibodies in this group of patients. Poil et al.^7^ reported the case of a 27-year-old female who presented with right-sided hemichorea as the first and only manifestation of SLE. The patient was also positive for antiphospholipid antibody and was treated with aspirin and hydroxychloroquine. The chorea resolved in 3 weeks and showed no recurrence during 6 months of follow-up. Abdalla et al^8^ described the case of a 14-year-old female who presented with generalised chorea and on evaluation was found to have lupus with renal and haematological involvement. She was positive for anti-cardiolipin Ig G and improved with pulse methylprednisolone, hydroxychloroquine, mycophenolic acid, and enoxaparin. In prior case reports where chorea has been the presenting feature of SLE, the onset of other clinical features of SLE has varied from being present during the initial evaluation of chorea to a few weeks after the onset of chorea.^9–13^

The exact pathogenesis of chorea in SLE may be multi-factorial involving 1) ischemia of small and large vessels in CNS mediated by anti-phospholipid antibodies and immune complexes, and 2) inflammatory neuronal injury secondary to complement activation, inflammatory cytokines, and increased permeability of blood-brain barrier causing autoantibodies to migrate to the intrathecal space. Antiphospholipid antibodies have a strong association with chorea and have been shown to cause toxicity to neurons and inhibit neuronal plasticity.^14^

With regards to the treatment of chorea in SLE, there is no consensus on the ideal management. In the analysis of 51 cases of chorea in SLE, Bruyn et al.^6^ has reported a good response with prednisone and haloperidol. However, there was no relationship between the dose of medications and duration of the choreatic episode. Reiner et al.^15^ retrospectively analysed the long-term outcome of chorea in patients with SLE or antiphospholipid antibodies. Among 30 patients, improvement of chorea was seen in 75% of cases with corticosteroids and 76% cases showed improvement with the use of neuroleptics. A minority of patients improved after treatment with a single agent like aspirin (n=3) or anticoagulant (n=1). Four patients who did not improve with the above-mentioned drugs responded to intravenous immunoglobulin (n=2) or plasma exchange (n=2). On follow up, eight patients had a relapse of chorea with a mean delay of 3.4 years. In a retrospective review of lupus-related chorea, Carvallo et al.^16^ found five patients with seven episodes of chorea. In 4 patients, moderate to high dose corticosteroids were combined with low dose dopamine antagonist due to generalised lupus activity and an early resolution of chorea was noticed. Cyclophosphamide was used in two of these patients due to the presence of proliferative lupus nephritis. **Table 1** shows the various drugs used for the treatment of chorea in SLE in the previously reported cases and the treatment outcome. The EULAR recommendations for the management of NPSLE state that in SLE-related chorea, symptomatic therapy with dopamine antagonist may be combined with antiplatelet agents in the case of anti- phospholipid positivity. Glucocorticoids and anticoagulation are considered in patients with generalised lupus activity and severe disease.^17^ Our patient, though serologically active did not have clinically active extra-CNS disease at initial presentation and hence was managed with a low dose of oral steroid and dopamine blocker.

## CONCLUSION

Chorea may be the first and only symptom of SLE. Though chorea has various differential diagnoses, SLE and antiphospholipid antibody syndrome should always be considered, especially in young females.
